# Artificial Intelligence in Dentistry: A Descriptive Review

**DOI:** 10.3390/bioengineering11121267

**Published:** 2024-12-13

**Authors:** Sreekanth Kumar Mallineni, Mallika Sethi, Dedeepya Punugoti, Sunil Babu Kotha, Zikra Alkhayal, Sarah Mubaraki, Fatmah Nasser Almotawah, Sree Lalita Kotha, Rishitha Sajja, Venkatesh Nettam, Amar Ashok Thakare, Srinivasulu Sakhamuri

**Affiliations:** 1 Pediatric Dentistry, Dr. Sulaiman Alhabib Medical Group, Rayyan, Riyadh 14212, Saudi Arabia; 2 Division for Globalization Initiative, Liaison Center for Innovative Dentistry, Graduate School of Dentistry, Tohoku University, Sendai 980-8575, Japan; 3 Department of Periodontics, Inderprastha Dental College and Hospital, Ghaziabad 201010, Uttar Pradesh, India; 4 Pediatric Dentistry, Sri Vydya Dental Hospital, Ongole 52300, Andhra Pradesh, India; 5 Preventive Dentistry Department, Pediatric Dentistry Division, College of Dentistry, Riyadh Elm University, Riyadh 13244, Saudi Arabia; 6 Department of Pediatric and Preventive Dentistry, Datta Meghe Institute of Medical Sciences, Wardha 442004, Maharashtra, India; 7 Therapeutics & Biomarker Discovery for Clinical Applications, Cell Therapy & Immunobiology Department, King Faisal Specialist Hospital & Research Centre, P.O. Box 3354, Riyadh 11211, Saudi Arabia; 8 Department of Dentistry, King Faisal Specialist Hospital & Research Centre, P.O. Box 3354, Riyadh 11211, Saudi Arabia; 9 Department of Basic Dental Sciences, College of Dentistry, Princess Nourah bint Abdulrahman University, P.O. Box 84428, Riyadh 11671, Saudi Arabia; 10 Clinical Data Management, Global Data Management and Centralized Monitoring, Global Development Operations, Bristol Myers Squibb, Pennington, NJ 07922, USA; 11 Department of Orthodontics, Narayana Dental College and Hospital, Nellore 523004, Andhra Pradesh, India; 12 Department of Restorative Dentistry and Prosthodontics, College of Dentistry, Majmaah University, Al-Zulfi 11952, Saudi Arabia; 13 Department of Conservative Dentistry & Endodontics, Narayana Dental College and Hospital, Nellore 523004, Andhra Pradesh, India

**Keywords:** artificial intelligence, dentistry, dental caries, oral health

## Abstract

Artificial intelligence (AI) is an area of computer science that focuses on designing machines or systems that can perform operations that would typically need human intelligence. AI is a rapidly developing technology that has grabbed the interest of researchers from all across the globe in the healthcare industry. Advancements in machine learning and data analysis have revolutionized oral health diagnosis, treatment, and management, making it a transformative force in healthcare, particularly in dentistry. Particularly in dentistry, AI is becoming increasingly prevalent as it contributes to the diagnosis of oro-facial diseases, offers treatment modalities, and manages practice in the dental operatory. All dental disciplines, including oral medicine, operative dentistry, pediatric dentistry, periodontology, orthodontics, oral and maxillofacial surgery, prosthodontics, and forensic odontology, have adopted AI. The majority of AI applications in dentistry are for diagnoses based on radiographic or optical images, while other tasks are less applicable due to constraints such as data availability, uniformity, and computational power. Evidence-based dentistry is considered the gold standard for decision making by dental professionals, while AI machine learning models learn from human expertise. Dentistry AI and technology systems can provide numerous benefits, such as improved diagnosis accuracy and increased administrative task efficiency. Dental practices are already implementing various AI applications, such as imaging and diagnosis, treatment planning, robotics and automation, augmented and virtual reality, data analysis and predictive analytics, and administrative support. The dentistry field has extensively used artificial intelligence to assist less-skilled practitioners in reaching a more precise diagnosis. These AI models effectively recognize and classify patients with various oro-facial problems into different risk categories, both individually and on a group basis. The objective of this descriptive review is to review the most recent developments of AI in the field of dentistry.

## 1. Introduction

Artificial intelligence (AI) is a technology that mimics human behavior using machines, with increased usage in numerous industries since 2020, including healthcare. It can replicate human intelligence and evolve based on retrieved information [1]. In the field of dentistry, clinicians collaborate with researchers to develop algorithms for measuring and analyzing clinical assessments, photographs, radiographs, and chart notes. AI can allow patients to regulate their care and to refine the accessibility to health information [2,3]. AI also focuses on neural networks modeled after human brains, forming a data processing system to address precise issues. AI is rapidly evolving, enabling robots to perform previously human-only tasks. Dentistry has recently begun using AI, leading to exceptional achievements in analyzing clinical dental data. Advances in AI demonstrate possible benefits for healthcare, such as improved decision making, fewer unneeded therapies, improved quality of life, and fewer postoperative problems. Machine learning seeks to forecast outcomes from a dataset without human involvement, whereas AI refers to a computer’s capacity to solve issues using data. Neural networks use artificial neurons to compute signals in a manner analogous to the human brain, whereas deep learning employs multiple computational layers to enhance detection. Data science is the process of examining and drawing conclusions from data, while big data is the practice of analyzing massive datasets in order to deliver precise consumer insights, and grasping the core components of contemporary AI systems is critical for gaining a comprehensive grasp of AI.

In engineering science, the field of AI studies how effectively processors understand calculations and how they may imitate patients’ cognitive abilities to behave sensibly and to efficiently perform tasks. An essential element of artificial intelligence, an intelligent network simulates the judgment processes of a patient’s knowledge and displays an experience as information or a standard inside the system. These recommendations and data may be used to resolve any concerns. In an intelligent network, a selection of dental patients is retained in a separate database called prototypes. The data form the basis for analysis, diagnosis, and recommendations and is taken from academic publications and human-subject-matter experts. The information is represented by combining the design of manufacturing rules with neural networks [4]. Artificial intelligence in dentistry can support the dentist in generating clinical decisions and assist in client diagnosis to provide the best care possible. Additionally, it could examine any unusual alterations to the oral mucosa. The application of intraoral scanners and cameras in dentistry offices nowadays has significantly aided the evaluation and scheduling of care [5]. The adoption of convolutional neural networks (CNNs) will also contribute to the simplification of diagnostic processes in dental offices. An artificial neural network (ANN), a statistical model or computer program, can mimic the neural network of the human brain to carry out cognitive functions like problem-solving [6]. In the healthcare industry, AI systems are often used to evaluate pictures by removing elements from specific images and carrying out thorough research. For example, AI systems may reduce the amount of effort the radiologist must spend diagnosing images and expedite treatment decisions by studying collections of chest radiographs and lung images obtained from patients with dental problems. Furthermore, while they are still modest compared with healthcare technology, technical developments in dentistry are becoming more noticeable [7]. Conventional digital dental techniques are utilized more often in typical treatment plans [8]. Artificial intelligence offers innovative decision-supporting technology in the field of dentistry. Focusing on artificial intelligence will only raise the level of treatment since people desire more extensive and detailed medical care.

## 2. Principles of Artificial Intelligence

**Figure 1:**
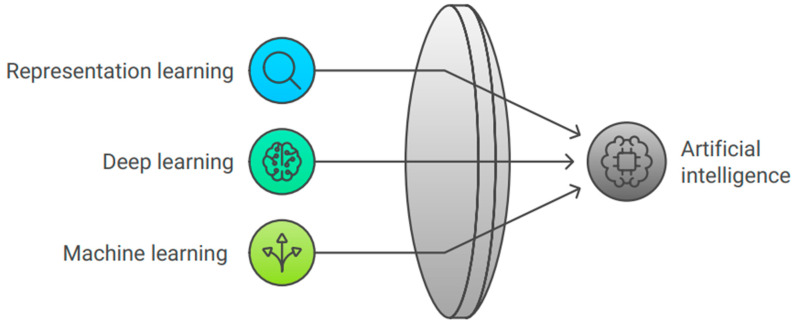
Principles of artificial intelligence.

Machine learning, representation learning, and deep learning are all included in the artificial intelligence paradigm, and Figure 1 represents their relationship with artificial intelligence.

### 2.1. Machine Learning

Machine learning (ML), which relies on similarities and interpretation rather than specific commands, is the philosophical study of the methods used by computer systems to conduct a particular job efficiently [9]. In the practice of machine learning, a statistical model is constructed based on sample data, which is more frequently referred to as “training data”. This model is then used to teach the machine how to make judgments without being explicitly programmed to complete the work [10].

### 2.2. Representation Learning

This is a machine learning (ML) form where the machine system learns the characteristics needed for categorizing the given input. It does not need the manual categorization of information as required by machine learning [11].

### 2.3. Deep Learning

Deep learning is a subcategory of machine learning that belongs to a bigger group of methods founded on artificial neural networks. Deep-learning frameworks have been used in various domains, including “deep neural networks, deep perception networks, recurrent neural networks, and convolutional neural networks” [12]. This encompasses drug manufacturing, radiological image interpretation, and histopathological condition identification, all of which have produced equivalent findings. In certain cases, superior outcomes are attained by specialists [12].

## 3. Application of AI in Healthcare

**Figure 2:**
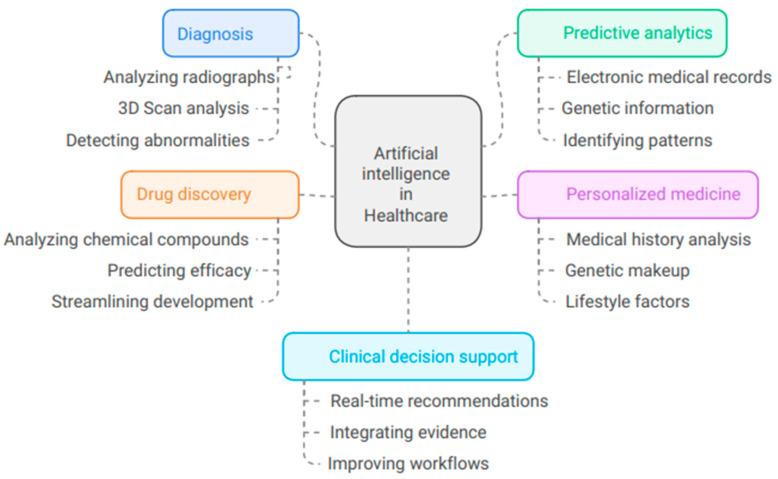
Illustrates the uses of artificial intelligence in health care.

AI is transforming the medical field through a variety of innovative applications (Figure 2).

### 3.1. Diagnosis

AI algorithms are adept at analyzing medical images like radiographs and 3D scans to detect abnormalities, aiding healthcare professionals in making more accurate diagnoses, which potentially lead to earlier interventions and improved patient outcomes.

### 3.2. Predictive Analytics

By using vast data like medical records and genetic information, AI can predict disease likelihood, enabling healthcare providers to anticipate patient outcomes and implement preventive measures, thereby improving patient care and resource allocation.

### 3.3. Personalized Medicine

AI is crucial in creating personalized treatment plans by analyzing a patient’s medical history, genetic makeup, and lifestyle factors, suggesting effective therapies that not only enhance treatment efficacy but that also minimize adverse effects.

### 3.4. Drug Discovery

AI is revolutionizing the drug discovery process by analyzing chemical compounds and predicting their therapeutic efficacy, thereby accelerating the pipeline and potentially bringing new treatments to the system faster.

### 3.5. Clinical Decision Support

AI systems offer real-time medical recommendations to doctors, enhancing decision-making, clinical workflows, and patient safety. They integrate updated information with patient data, revolutionizing diagnosis, treatment, and patient management. As technology evolves, AI integration in healthcare is expected to expand, leading to further improvements in patient care and outcomes. This demonstrates the potential to revolutionize healthcare delivery and improve patient outcomes.

## 4. Use of Artificial Intelligence in Dentistry

**Figure 3:**
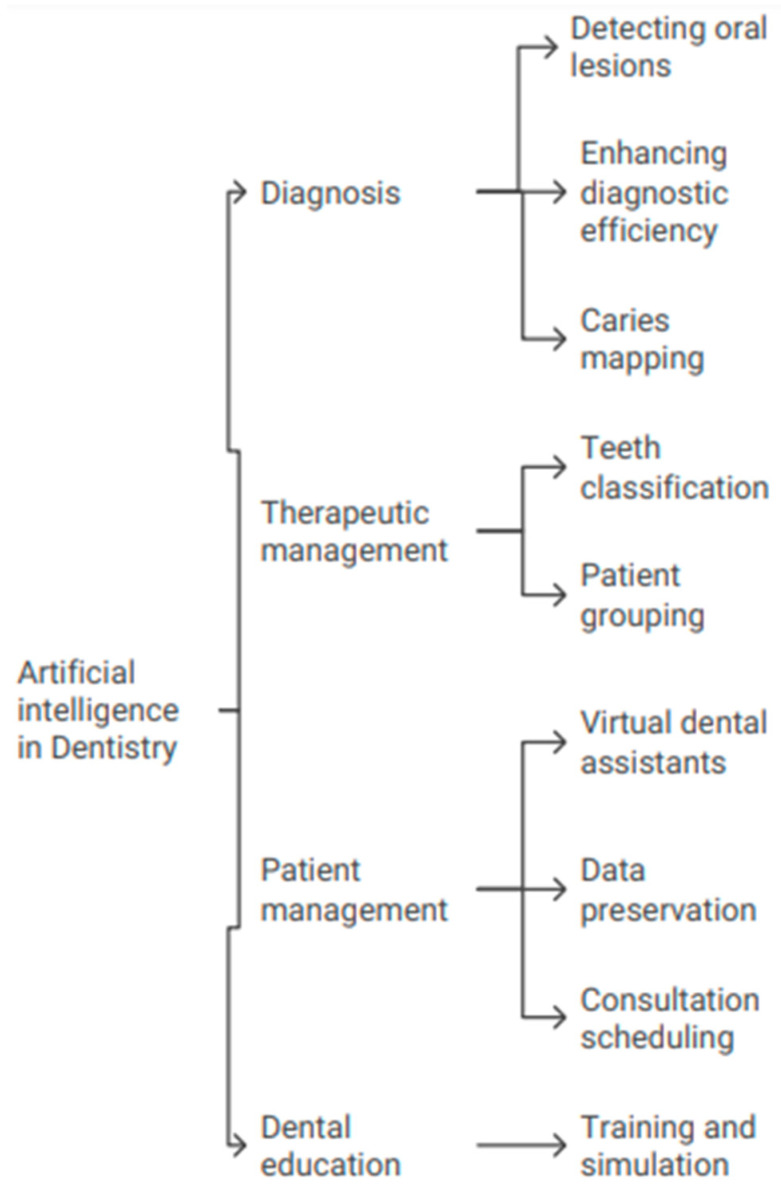
Illustrates the use of artificial intelligence in dentistry.

Dentistry is transforming thanks to AI, which is improving diagnosis accuracy, personalizing treatment regimens, and organizing data more efficiently [9,10]. Different areas of dentistry are making use of advances in AI, especially machine learning frameworks like CNNs. These AI tools offer many benefits, such as finding diseases earlier. Nevertheless, overcoming obstacles related to data management, processing capacity, and ethical considerations is necessary for the successful integration of AI in dentistry [13]. The use of artificial intelligence dentistry has been illustrated in Figure 3.

### 4.1. Artificial Intelligence in Diagnostics

Artificial intelligence can be applied as a helpful technique in the detection and therapy of oral cavity diseases, as well as in detecting and categorizing suspected changed mucosa experiencing premalignant and malignant alterations. AI has completely transformed the discipline of diagnostics [14]. The program allows users to upload images of lesions using essential smartphone applications and correlates these photos with a vast amount of globally accessible data to arrive at diagnoses. As a result, medical professionals can emphasize the need for an appointment when it is required, and patients have quick access to the opinions of specialists [15]. Using artificial intelligence to screen for and to diagnose worrisome oral lesions as premalignant or malignant lesions may be helpful. AI provides the clinician with subjective and statistical data based on the data inputted by the therapist, thereby enhancing the physician’s efficiency in timely identification, prognosis, and therapeutic management [16]. Loading each input radiograph into the AI modal neural network led to the development of caries possibility mapping and construction possibility mapping. Software was produced to view identified dental caries as a region on the bitewing radiograph and objectively represent the proportion of dental caries. This was accomplished by displaying the program’s output in numerical form [17].

### 4.2. Artificial Intelligence in Therapeutic Management

In a classification of teeth to “gold standard” based on the National Institutes of Health (NIH) general agreement transformation plan, which explains well-established requirements for the separation of third molars, it was discovered that neural networks, when ideally trained on lower third molars, have elevated particularity and responsiveness comparable to specialized consultation [18]. It may also be used to decide if separations are required before receiving orthodontic therapy. Furthermore, depending on their immunological system response pattern, it may be utilized to divide patients into groups with aggressive and chronic periodontitis [19].

### 4.3. Artificial Intelligence in Patient Management

Virtual dental attendants may handle patients in the dental operatory with fewer human inaccuracies and more precision while using fewer resources. Artificial intelligence technology aids in more effective data preservation, and extensive digital information may be utilized afterward to provide individuals with dental problems with the best care possible as and when needed [20]. It may also be used to gather all necessary dental data and to manage consultations, licensing, and scheduling. Scheduling consultations for individuals with dental problems at the clinic, gathering the patient’s complete health and oral history, handling financing, and aiding the dentistry dentist with a suitable assessment and therapeutic plan may all be possible [21]. Additionally, it operates by alerting the dental expert about the patient’s healthcare record and routine habits, such as their usage of alcohol or cigarettes. In this manner, a digital record of each patient can be produced, aiding the dentist in illness therapy and evolution. Additionally, it will help with patient follow-up and virtual critical medical consultation [22].

### 4.4. Artificial Intelligence in the Dental Education System

Clinical dentistry is crucial for dental students to develop their abilities and improve patients’ utmost care. Preclinical operational training has historically included conceptual and hands-on instruction for dental learners. In recent years, intelligent education and dental training teaching systems have included artificial intelligence [23]. Through these innovations, real-world operations may be virtually recreated in three dimensions, allowing for the assessment of clinical and surgical methods. Before managing actual clinical situations, students may participate in practice rounds as often as necessary to become experts in the field and to lower the danger of iatrogenic harm. This training approach is more effective, affordable, and trustworthy [24].

## 5. Artificial Intelligence in Clinical Dentistry

### 5.1. Artificial Intelligence in Pediatric Dentistry

The adoption of artificial intelligence is gaining traction across all industries and is also breaking new ground in pediatric dentistry. Artificial intelligence offers a diverse range of promising solutions, starting from investigation and diagnostic testing and extending to behavioral administration, pain management, prosthodontic tooth mobility, and regenerative dental care [25]. These implementations hold the potential to revolutionize the learning and practice of modern dental care. The integration of AI with other emerging innovations, such as virtual reality (VR) in the form of a virtual education environment, has the potential to assist more children and to provide a customized learning experience [26]. A recent systematic review states that AI and ML are mostly used to create tools for checking people’s health, like the Children’s Oral Health Score (COHSI) and the Referral for Treatment Needs of Oral Health (RFTN); they are also used to find extra teeth, plaque, and to assess the suitability of fissure sealants; to predict early childhood cavities; to guess dental age; to find submerged teeth; and to find teeth that are erupting in the wrong place [27]. A systematic review categorized five clinical applications of AI, which include assessing genetic risk, analyzing dental characteristics and the relationship between the upper and lower jaws, detecting hypernasality, performing cleft-lip palate surgery, and diagnosing and forecasting oral clefts. AI aids in clinical decision making, cephalometric analysis, and treatment prediction [28]. Dental infections are prevalent chronic childhood illnesses and are a serious public health concern for young people of all ages in industrialized and emerging cultures. These disorders may severely impact a child’s dental health and overall welfare. Thus, their quick identification and management are essential. Although dental illnesses are inherently controllable, rapid and precise potential risk detection may be crucial for creating prevention strategies against expensive diseases. Researchers have established caries probability estimation techniques to identify warning indicators for oral illnesses like dental caries, potentially leading to the development of preventative interventions that could improve medical therapy [29]. The most popular cavity risk screening models are the cariogram, the “Caries Risk Assessment Tool (CAT)”, and caries treatment by hazard analysis. Reports recognize the cariogram as a reliable tool for predicting cavity danger, with a sensitivity range of 41–75% and a specificity range of 65.8–88% [30]. Dental cavities are a persistent, complex, and sugar-dependent bacterial infection of the teeth. The disease arises from a discrepancy between the processes of demineralization and remineralization. Certain caries-causing bacteria, the host’s preparedness, a diet rich in unfermented sugars and carbohydrates, and the duration of the host’s exposure to this potentially cariogenic diet are the main causes of this difference. Modifiable variables such as socioeconomic status, lifestyle, food habits, and inadequate cleanliness can also contribute to the spread of the disease [31]. Al-Jallad et al. [9] developed an AI-processed application to detect caries using children’s photos. The Caries app received a score of 78.4 on the System Usability Scale (SUS), indicating excellent acceptance. This app enables parents to use smartphones to take pictures of their children and can be a useful tool in preventing caries.

### 5.2. Artificial Intelligence in Prosthodontics

AI assists prosthodontics by utilizing computer-assisted layout and fabrication software to ensure the accuracy and suitability of crowns and removal appliances for tooth replacement. However, researchers are using AI to instantly develop innovative dental reconstructions for personalized suitability, perfect activity, and improved structural systems, thanks to the advancements in confrontational conceptual networks [32]. The software will likely guide the dentist through the entire process of creating a digital imprint and aid in creating a high-quality impression through the application of artificial intelligence. This will significantly enhance the patient’s perception of prosthodontics. It assists in the analysis of arch patterns and contributes to the construction of detachable partial or complete prostheses [33]. Because of advances in virtual reality, the procedure of delivering esthetic prosthetics and attending to patients’ requirements has become much more straightforward. The client can attempt a digital prosthesis with AI systems and simulated reality [34]. The patient can customize these virtual prosthetics to their preferences until they are satisfied. These specifications precisely sculpt the finalized prosthesis [35]. The application of AI to the study of implantology has made it possible to build prostheses in an accurate and automated way. Additionally, it has simplified the process of determining the optimal placement of implants. The tongue-driving technology’s artificial intelligence is capable of monitoring tongue movements in the oral cavity and responding to instructions [36]. Alharbi et al. [37] developed four ML algorithms to identify the necessity of dental implants. They used the improved AdaBoost algorithm for dental implant prediction. Among these models were the AdaBoost method, the Random Forest, the Bayesian network, and the enhanced AdaBoost algorithm. It is clear from analyzing the findings that the built machine learning performed exceptionally well. This study’s modified AdaBoost algorithm achieved 91.7% accuracy in predictions, which is far higher than the other methods tested, and yielded much better performance overall [37]. To examine the use of AI in implant-supported therapy, Bornes et al. [38] conducted a comprehensive literature analysis of sixteen papers. Thirteen publications demonstrated the evolution of AI algorithms for ML, DL, and CNNs. To better diagnose damage, optimize implant placement, and improve oral rehabilitation, most studies have used 2D imaging to identify implants.

The combination of AI algorithms and omics sciences has resulted in the creation of bioinformatics tools, which not only reduce misdiagnosis but also enable the prediction of potential outcomes. Desktop manufacturing and design processes, specifically “computer-aided design (CAD/CAM)”, have found their way into standard laboratory and hospital practice. One novel concept that is beginning to surface as dentistry digitalization progresses is AI [7]. Artificial intelligence may also be utilized to evaluate the debonding of dental repairs based on flat imaging. In removal prosthodontics, convolutional neural networks (CNNs) may classify dental arches. Building denture teeth in edentulous patients that meet both functional and esthetic requirements is never simple for dental professionals. Machine learning in CAD/CAM software may restore sound inter-maxillary connections by placing the teeth correctly. When it comes to difficult esthetic situations like having several front teeth or only one central incisor, AI may help with proper color matching. Implant placements may be quickly detected by intraoral detectors and entered into CAD software in implant dentistry. AI can potentially enhance dental implant conception and implementation [8].

### 5.3. Artificial Intelligence in Orthodontics

**Figure 4:**
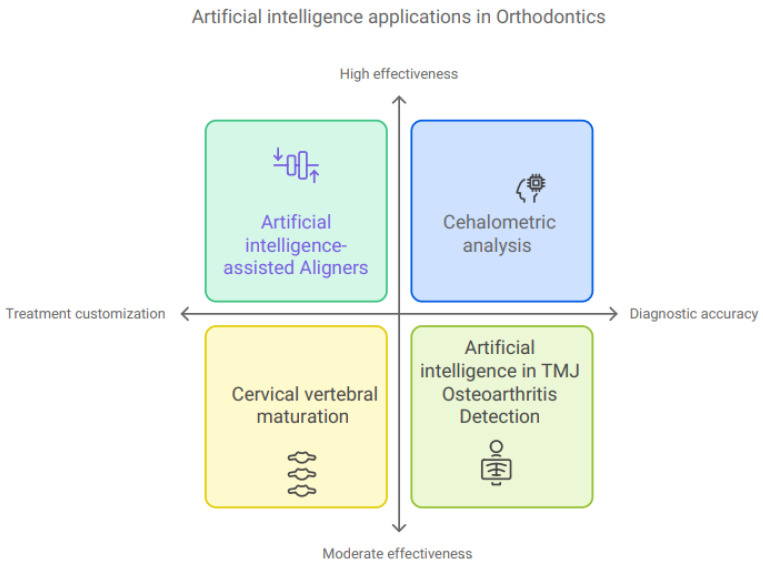
Illustration of the use of artificial intelligence in orthodontics.

The use of AI to customize orthodontic treatment is one of the most recent innovations that has attracted an abundance of interest (Figure 4). Using accurate 3D photographs and simulated representations, it is simple to 3D-print the appliances [39]. Using a patient’s unique set of dental information, this system intelligently computes the required adjustments, finds the optimal force, and locates the pressure sites [40]. The AI-assisted aligners not only provide exact therapy implementation but also assist in evaluating the progression of the therapy. They promise to shorten the time required for medicines while simultaneously reducing the number of necessary consultations [41]. Researchers have found that cephalometric analysis is more reliable and consistent than manual analyses, which depend a lot on how well the operator can identify landmarks and often show big differences [42,43,44,45]. Accurate cephalometric analysis results depend on precise and consistent landmark identification. Artificial intelligence has shown effectiveness in identifying cephalometric landmarks in multiple experiments. The most common method for cephalometric analysis is lateral radiography; however, CBCT has recently seen a renaissance with AI developments [46]. In cervical vertebral maturation evaluations, models based on CNNs achieved accuracy rates of over 90%, as reported by Seo and colleagues. [47] The authors reported that, as a result, lateral cephalometric radiographs may be useful for the automated identification of a child’s bone maturity status. It is crucial to proceed with caution when analyzing AI outcomes in cervical vertebral maturation evaluations. There have been other studies that have found significant differences, especially during the growth peak and other critical times of orthodontic therapy when the accuracy is typically lower [48,49]. Recent research findings demonstrate the exceptional diagnostic capabilities of AI in detecting and staging temporomandibular joint osteoarthritis [50,51,52]. Researchers used several imaging techniques, including panoramic radiographs, cone-beam computerized tomography, and magnetic resonance imaging, to demonstrate the feasibility of an automated, comprehensive evaluation of joint morphology. Researchers believe that using AI systems for diagnostic imaging of the temporomandibular joint will make it easier to detect arthritis early and to find the best treatment for each person. Several reviews and meta-analyses in this area have demonstrated the models’ moderate to high accuracy in identifying temporomandibular joint osteoarthritis [53,54,55].

### 5.4. Artificial Intelligence in Oral Medicine and Radiology

**Figure 5:**
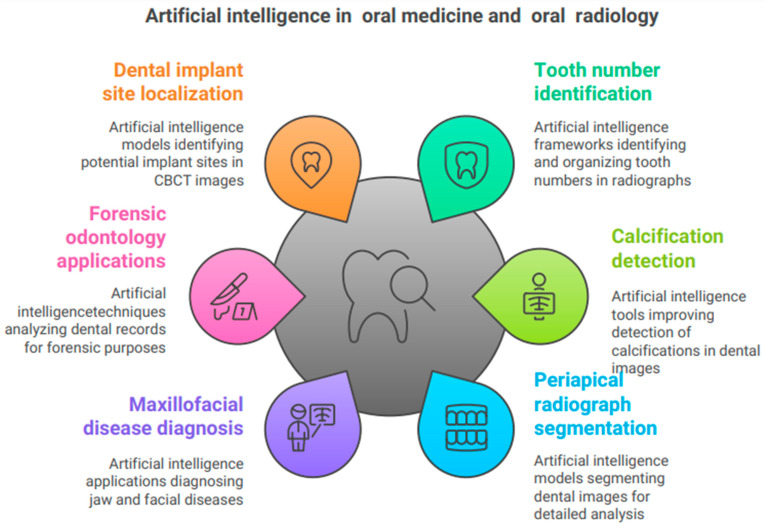
The use of artificial intelligence in oral medicine and radiology.

Kabir et al. [56] created an AI framework to recognize tooth numbers in panoramic and intraoral radiographs and to organize full-mouth radiographs according to an FMS layout template. The procedure has two stages. The initial step involves assigning a tooth number to each tooth in periapical and bitewing radiographs, followed by organizing the intraoral radiographs in a full-mouth series (FMS) configuration. The study’s primary findings encompass the introduction of a model that surpasses earlier models in both specificity and sensitivity. Connecting this model with other dental diagnostic models and EHR systems enables the validation of clinical charting through deep-learning-based clinical reporting [56]. Song et al. [57] evaluated the efficacy of AI-based techniques in identifying soft-tissue calcifications. The study randomly chose 60 participants with sialoliths and carotid artery calcifications, respectively. The research examined three forms of calcification: carotid artery calcification, sialolith calcification, and lymph node calcification. The objective was to evaluate the impact of the AI system on general dentists’ visual interpretation skills. As expected, using AI increased the number of calcification corrections performed by both general dentists and oral medicine radiologists. However, reading time increased for general dentists while it decreased for oral medicine radiologists. The results suggest that, when effectively employed, panoramic imaging can serve as a valuable screening instrument for diagnosing other disorders [57]. Ari et al. [58] conducted an assessment of periapical radiographs utilizing a U-Net-based artificial intelligence model grounded in convolutional neural networks. The study’s main results showed that the deep-learning models were able to separate the periapical test images made by the AI model, with an F1 score of up to 80% for sensitivity and accuracy. The study’s limitations include its reliance on a single radiographic machine for imaging, the absence of an external dataset, the lack of observers with diverse backgrounds, and the omission of multiple CNN models. This AI model, which is based on the U-Net architecture, made it more accurate in differentiating cavities, crowns, dental fillings, dental pulp, periapical lesions, and root canal fillings in pictures of the back of the tooth [58,59]. Baydar et al. [59] evaluated bitewing photos using AI applications trained with deep-learning techniques, thereby establishing the trustworthiness of the U-Net model. The diagnosis of caries had the lowest success rate, but other dental diagnoses, such as dental crowns, restorations with filling material, and root canal fillings, attained a success rate of 95% [59]. The study by Hung et al. [60] emphasizes the application of deep learning and radiomics in CT and CBCT for the diagnosis and management of maxillofacial disorders. They suggest different ways to automatically find, separate, and label jaw cysts, tumors, problems with the salivary glands, the temporomandibular joint, the maxillary sinuses, broken jaws, deformities of the dentomaxillofacial area (Figure 5), and problems with the mandible. Shahnavazi and Mohamadrahimi [61] devised a deep-learning algorithm that autonomously detects mandibular fractures and injuries, evaluating its efficacy against that of general dentists. This research utilized a dual-phase deep-learning system. The authors initially employed a Unet model to segment the mandible as the area of interest. The authors employed a model known as the Faster Region-Based Convolutional Neural Network (Faster R-CNN) to analyze panoramic radiographs and to identify fractures in the mandible, along with their specific locations. The authors assessed the categorization model’s accuracy at 91.67%. On average, the model outperformed humans in diagnostic accuracy (91.67 vs. 87.22 ± 8.91) and sensitivity (82.22 ± 16.39) [61]. Mohammad et al. [62] conducted a scoping assessment of 28 papers to ascertain the uses of artificial intelligence in forensic odontology. Four categories delineate the prospective applications of AI technology in forensic odontology: (1) analysis of human bite marks, (2) sex determination, (3) age estimation, and (4) dental comparisons. This powerful tool can help solve the world’s problems by providing enough datasets, the right way to use algorithmic architecture, and the right way to distribute hyperparameters that assist the model to make accurate predictions [62]. Alsomali et al. [63] created an AI model that autonomously locates markers in radiographic stents to determine prospective implant sites within CBCT images. The testing dataset comprised 50 picture segments, with 193 including gutta-percha (GP) markers and 2284 lacking them. That study introduced the inaugural AI model designed for the identification of GP markers utilized to pinpoint potential dental implant locations within CBCT images. The existing system successfully identified the majority of GP markers; nevertheless, it produced 2.8% false positives and overlooked 17% of cases. Relying just on axial images to train an AI program is insufficient to ensure proper performance of the AI model [64]. Choi et al. [65] developed an AI tool that uses deep learning to automatically find natural teeth and dental treatment patterns in dental panoramic radiographs (DPRs). This makes DPRs more useful as identifiers of people. The researchers employed a pre-trained object identification network that utilized an efficient Det-D3 convolutional neural network to identify natural teeth, dental treatment patterns, and tooth numbers. The objective metrics for average precision utilizing dental panoramic radiography were 99.1% for natural teeth, 80.6% for prostheses, 81.2% for treated root canals, and 96.8% for implants. This study demonstrated that convolutional neural networks excel at autonomously identifying tooth numbers and locating natural teeth, dentures, implants, and treated root canals. The growing focus on diagnostic techniques, such as digital RVGS/IOPA, 3D scans, and CBCT, is facilitating the gradual integration of AI into dental radiology. It is feasible to acquire and assess extensive data to create an AI that facilitates prompt diagnosis and therapeutic scheduling [66].

### 5.5. Artificial Intelligence in Periodontics

In recent years, dentistry and sophisticated education systems have incorporated AI into their instruction and learning systems. These technologies can create virtual realities, allowing the modeling of functional processes in three dimensions and, thereby, facilitating access to medical and therapeutic methods [67]. Before the actual management of natural medical settings, students may participate in the training exercises as many times as necessary until they have developed experience in the relevant matter. This lowers the likelihood that they will cause iatrogenic injury [68,69]. This kind of instruction is more effective, not to mention more cost-effective and trustworthy. Periodontal hazard analysis has effectively utilized AI [69]. The approach incorporates various factors such as aging, bleeding during probing, the average depth of the pocket under investigation, the presence of root plaque, and the degree of lateral bone deterioration observed on dental radiographs [70]. Periodontitis is the sixth most prevalent disease worldwide. Microbes and the host cause inflammation that results in the loss of alveolar bone and periodontal attachment, potentially leading to tooth loss [71]. Periodontitis remains a significant oral health challenge, with high rates of untreated disease exhibited among certain high-risk and disadvantaged groups [72]. An estimated 20% to 50% of the world’s population suffers from this chronic dental disease [73], and its incidence positively correlates with age. However, there is a lack of standardization relating to periodontitis diagnosis and management, resulting in instances of undiagnosed and untreated oral disease [74,75]. Progressive approaches to care, such as incorporating AI technology into dental practice software, can assist dental providers in standardizing the diagnosis of periodontitis and increasing treatment acceptance by improving patients’ health literacy and understanding of their periodontal condition. New technology and innovative approaches to care, such as incorporating AI technology into dental practice software, can improve patient education methods, facilitate clinical decision making, enhance clinical efficiency, and promote intra- and interprofessional collaboration [76]. Some types of AI-powered radiograph analysis can measure from the cementoenamel junction (CEJ) to the crestal bone, which assists dentists in their clinical decision making and diagnostic consistency. The use of AI can also help improve patient engagement, thereby facilitating optimal health outcomes and health service utilization [77]. Further, improving health literacy via patient education enables patients to better understand their condition, which can further reduce treatment costs by improving health behaviors [78]. Researchers currently find AI useful in diagnosing periodontal disease, predicting a specific condition, and creating treatment plans tailored to each patient [77,78]. Also, algorithms and AI-enhanced software can help dentists improve patient communication and demonstrate the necessity for treatment. Research shows that one AI model can accurately diagnose periodontal disease in premolars and molars with accuracy rates of 81% and 76.7%, respectively. Another model can identify periodontitis by analyzing a patient’s subgingival plaque to distinguish microbial profiles [79]. Nakano et al. [80] used deep learning to detect oral malodor from microbiota with a predictive accuracy of 97%. Danks et al. [81] used a deep neural network to measure periodontal bone loss by analyzing periapical radiographs. The system achieved a total percentage of correct key points of 89.9%. Tonetti et al. [70] utilized a deep-learning model to identify and quantify periodontal bone loss in panoramic images, subsequently aiding in the staging of periodontitis.

### 5.6. Artificial Intelligence in Oral and Maxillofacial Surgery

**Figure 6:**
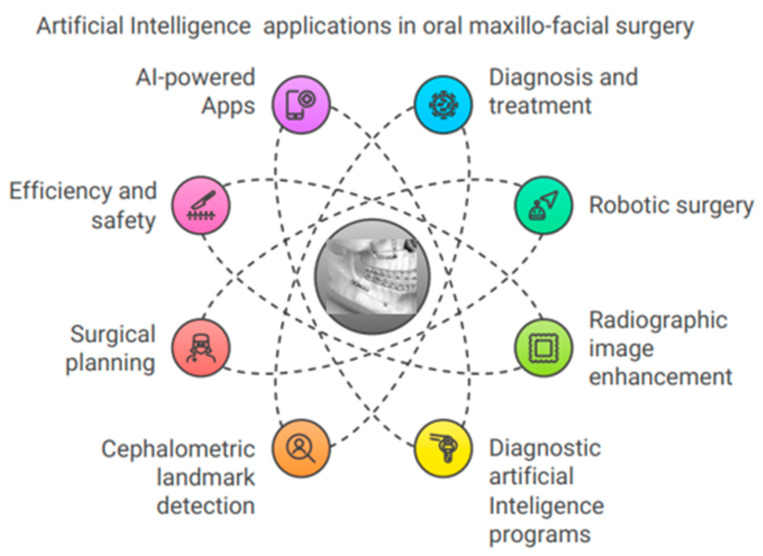
The use of artificial intelligence in oral maxillofacial surgery.

The most significant utilization of AI in dental surgery is the advancement of robot operations, which involve the recreation of human body movements and cognition (Figure 6). In medical settings, image-guided cranial functions can be used to successfully place dental implants, remove tumors and foreign bodies, conduct investigations, and perform tasks on the temporomandibular joint [82,83]. Even when carried out by skilled surgeons, close examination of oral surgical treatments reveals a much-increased level of reliability compared with the freehand approach. In contrast, there was no noticeable difference between students and professional physicians in terms of the outcomes [84]. Documentation generally shows shorter operation times, more intraoperative precision, and gentler handling around fragile tissues. Image guidance makes it feasible to perform a complete surgical resection, which might reduce the need for further revision procedures. Currently, multiple robotic physicians can perform semi-automated surgical operations with increasing efficiency, all under the supervision of a qualified surgeon. Advances in AI have enabled this transformation in surgery [85]. Clinical expertise helps orthognathic surgeons create complete treatment strategies that improve outcomes [86]. When designing and building splints, surgeons use CT or CBCT models to automatically register 3D craniomaxillofacial features [87,88]. Thus, three-dimensional assessments of hard- and soft-tissue movements before orthognathic surgery can guide technique selection. Due to defects and scar tissue, cleft patients’ soft tissues behave differently from those of non-cleft patients, making treatment helpful [89]. Software using AI may identify landmarks, analyze quick digital cephalometric data, advise healthcare decisions, and forecast treatment outcomes. Presurgical orthopedics, speech pathology detection, and the prediction of cleft lip and palate surgery outcomes all employ AI. The results have shown 85–95.6% model accuracy [90]. AI models predict perioperative blood loss, systemic infections, and orthognathic surgery [90,91,92]. Hong et al. [93] developed 75% of the cephalometric landmarks that have been crucially useful in orthognathic surgeries. This was true even when orthodontic brackets, surgical plates, screws, fixed retainers, genioplasty, and bone remodeling were in place. A CNN model using lateral and frontal cephalograms diagnosed orthognathic surgery cases with 94.4% accuracy [94]. Jeong et al. [95] showed that deep-learning CNNs can identify surgery patients based on frontal and lateral facial photos. A study by Tanikawa et al. [96] looked at AI’s ability to guess 3D facial shapes after orthodontic and orthognathic surgery. Researchers found that AI systems capable of guessing post-treatment face morphology are both safe and effective.

### 5.7. Artificial Intelligence in Endodontics

**Figure 7:**
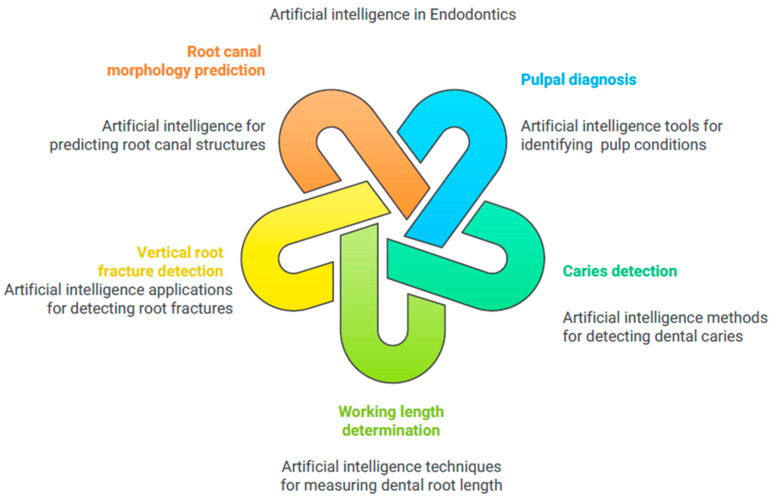
The use of artificial intelligence in endodontics and conservative dentistry.

AI has shown promise in distinguishing pulpal diseases using radiographs, but radiographic assessment has limits. Consideration should be given to both clinical and radiographic investigations in conjunction with pulp and periapical tests. This integrated diagnostic strategy ensures a complete investigation, improving clinical pulpal diagnoses. According to research, AI has considerably improved pulpal diagnosis (Figure 7). Based on panoramic radiographs, Tumbelaka et al. [97] employed an ANN to distinguish normal pulp, pulpitis, and necrotic pulp. Schwendicke et al. [98] implemented an AI-based convolutional neural network for caries detection on bitewings. The researchers applied the AI tool to 29,011 teeth without caries and 19,760 teeth with caries lesions, training it on 10%, 25%, 50%, or 100% of the dataset. The major outcomes of the study were that the AI-based tool proved to be cost-effective and highly accurate in detecting caries. This was the very first study to assess the value of training data for AI applications in dentistry. Qayyum et al. [99] proposed deep-learning techniques for caries detection in dental radiographs. People widely use dental radiographs for caries detection, but the annotation process of these images is costly and time-consuming. They presented a dataset of 141 images using a semi-supervised learning model, which enabled high-accuracy caries evaluation. This model achieved a performance improvement of 6% in pixel accuracy compared with self-supervised learning. According to Karobari et al. [100], common applications of AI tools include tracing the apical foramen, measuring the working length, detecting periapical lesions, and predicting appropriate treatments. AI methods such as CNN-based DetectNet with DIGIT version 5, Pyramids Attention Convolutional Neural Network (FPACNN), and machine learning were used to detect vertical root fractures. This study illustrates the AI tools’ superiority compared with conventional techniques. Other AI tools, such as neural network models and DL-based computer vision techniques, were used to detect the apical foramen and to predict root canal morphology. Digitizing direct-reading radiography improved pulpal diagnosis validation in their 20-tooth trial. Zheng et al. [101] used CNNs like VGG19, Inception V3, and ResNet18 to diagnose deep caries and pulpitis on panoramic radiographs. They examined 844 panoramic radiographs and discovered that a multi-modal CNN named ResNet18, in conjunction with clinical factors, enhanced the diagnosis of deep cavities and pulpitis. Endodontic treatments need accurate working length determination, which AI has improved. Saghiri et al. [102] employed an artificial neural network (ANN) in conjunction with a Perceptron model to analyze the panoramic radiographs of 50 single-rooted teeth, utilizing radiograph features to identify the small apical foramen. The study found that the ANN model improved the radiography working length determination accuracy. Another study by Saghiri et al. [103] investigated the accuracy of the ANN in cadaver models by positioning the file concerning the apical foramen. The study found that the ANN outperformed endodontists in working length determination using human cadavers with 50 single-rooted teeth. AI, particularly ANNs, may be able to assess working length more accurately than humans in some cases.

Qiao et al. [104] used multifrequency impedance to measure the working length L with ANNs. They incorporated impedance ratios, tooth types, and file properties into the circuit system. Although the number of examples was not given, the multifrequency impedance approach employing ANNs enhanced the working length measuring accuracy and robustness. These studies show that AI could revolutionize endodontic working length determination, providing more accurate and dependable results. A study created and compared two independent CNN algorithms in estimating the number of distal roots of mandibular first molars on panoramic radiographs [105,106]. Fukuda et al. [107] suggest using a CNN to detect vertical root fractures in panoramic radiography. Shah et al. [108] developed a method to automatically detect, quantify, and locate vertical root fractures in high-resolution CBCT (hr-CBCT) scans. In a different study, periapical radiographs and CBCT images were used to teach a neural network how to find vertical root fractures in teeth that were whole or had roots filled in [109,110]. The authors found that CBCT fracture detection of roots is more specific, accurate, and sensitive than 2D radiography. Khanagar et al. [111] conducted a systematic review and reported on the use of AI models in endodontics. The major outcome of the study was that the CNN-based AI models demonstrated excellent efficiency in diagnosing pulpal diseases and working length. Asiri and Altuwalah [112] reported that AI-based networks such as CNN, ANN, and DCNN have been a significant aid for diagnosis and treatment planning. Teleassistance has successfully used these models. These neural networks will be valuable tools for dental specialists to diagnose effectively.

### 5.8. Artificial Intelligence in Forensic Odontology

Artificial intelligence (AI) is a scientific advancement that has seen significant use in forensic medicine. It successfully identifies the biological age and gender of healthy and unwell patients with dental problems. Moreover, it has the potential to evaluate tooth marks and to predict mandibular anatomy [113]. The field of dentistry stands to gain from some of the most exciting applications of AI. A substantial change occurred in the dental chair, which went from a physiological, hydrostatic pressure seat with a mechanical compressor to an electronic one with various sensors connected. The dental chair is an essential component of the dental office. The most recent advancement in dental technology is a voice-controlled chair that eliminates the dentist’s need for manual tasks [114]. Voice commands complete all tasks. Soon, dentist chairs will be capable of monitoring patient’s vital signs, anxiety level, weight, and the duration of the procedure while also consoling the patients and alerting the operating physicians if any differences are observed. This will make dental procedures much more efficient. This is due to the fact that every intellectual mind is currently dedicated to the advancement of AI. One of the most creative uses of AI is bioprinting. The sector of “bioprinting”, which enables the generation of living cells and organs in consecutive thin layers of cells, is one of the most innovative applications of AI. One day, we may apply this technology to recreate hard and soft tissues that have failed due to pathophysiological or inadvertent reasons [115].

## 6. Discussion

AI has several potential uses in dentistry that may transform current behavioral dental practices. Additionally, machine learning algorithms will continue to advance with the aid of huge databases. Another area where machine learning algorithms are gaining traction is the mobility of adolescent orthodontic teeth with personalized AI-driven equipment. These appliances would be more popular with the younger population. The field of dentistry has previously used AI-enabled regenerative dentistry and computer-aided design and production technologies [116]. The condition of permanent teeth and gums is essential to general wellness and quality of life. The primary goal of dentistry is to achieve the highest possible level of dental hygiene in young adolescents by concentrating on diagnosing, providing therapy, and avoiding a wide range of oral illnesses, beginning in infancy and continuing through early childhood. Dental caries, pulpal and periapical lesions, gingivitis, and other problems, such as dental trauma and inadequate dental hygiene, are among the most prevalent oral illnesses impacting adolescents. Chronic oral conditions, particularly tooth caries, may make treatment more difficult, cause discomfort, reduce masticatory performance, or cause asymmetrical mastication [117]. This may potentially lead to impaired facial formation, which would contribute afterward to malocclusion and orofacial abnormalities. Studies also reveal a connection between severe dental caries during childhood and more extensive dental caries in adulthood. Technological improvements have led to the extensive use of innovative AI-based apps for the identification, diagnosis, and prognostic forecasting of dental disorders [118]. This study sought to address the challenges associated with assessing facial structure in cleft lip and/or palate patients as a component of a therapeutic outcome evaluation. For this purpose, a face detector and CNNs that had previously been trained on facial appearance were given facial scans of healed cleft lip and/or palate patients and standards. This finding demonstrates that the existing panel-based assessments of face beauty have dispersion-related problems and are essentially inaccessible to sufferers. Although the recent findings suggest that significant interchanges with AI models are required to better understand the influence of cleft characteristics on face attraction, AI might reveal itself as a useful method for describing facial beauty [119]. A recently developed deep-learning method classified temporomandibular joint osteoarthritis using a 3D interface model, which included 250 auricular parameters at varying levels in the training database. Once the software receives a fresh patient’s condyle interface model, the Slicer Shape Variation Analyzer (SVA) component should be able to categorize the degree of rheumatic deterioration of a condyle into five categories of morphological variance. Training on high-spatial-image data with architectural form characteristics enhanced the design of the present neural network [120]. The study aimed to develop an AI framework based on deep learning to identify plaque on baby teeth and to evaluate the model’s diagnostic efficacy. Compared with a skilled dental clinician, the AI model demonstrated medically satisfactory accuracy in identifying dental plaque on primary teeth. This result shows the promise of such uses of AI in enhancing children’s dental hygiene [121]. This study assessed the application of a deep-learning method for automatically identifying and counting permanent teeth as shown on transverse radiographs. The AI approach proved effective in identifying and cataloging children’s decaying teeth as shown on panoramic radiographs. High rates of sensitivity and accuracy were observed. The estimates for the F1 score, sensitivity, and accuracy were 0.9804, 0.9571, and 0.9686, respectively [122]. One of the main reasons people undertake orthodontic therapy is for an improved facial appearance. AI is utilized to assess the influence of dental alignment on various alterations, such as the application of glasses, jewelry, or lipstick on the beauty of the face and the estimation of age. Similar to applying lipstick, maintaining dental symmetry enhances facial beauty, but it does not significantly affect the estimated age. Although wearing glasses significantly affects one’s appearance, this impact fades with age [123]. Ethical frameworks and regulatory compliance are crucial for balancing innovation and patient rights. This involves securing informed consent, upholding patient autonomy, and safeguarding data integrity [124]. Emerging privacy-preserving methods, such as federated learning, present effective solutions for safeguarding data privacy while facilitating advancements in AI [125]. The incorporation of AI in dental operatories poses considerable challenges with regard to patient consent, data privacy, and the potential biases inherent in AI algorithms. The significance of these issues lies in their influence on patient trust, ethical standards, and the efficacy of AI-driven healthcare solutions. Ensuring strong data privacy and mitigating biases are critical for the ethical implementation of AI technologies in dental clinical settings. This document examines the challenges and explores potential solutions. Data privacy is essential, with regulations such as Health Insurance Portability and Accountability (HIPAA) and General Data Protection Regulation (GDPR) directing the safeguarding of sensitive patient information. Encryption, anonymization, and differential privacy are essential techniques for data protection [125,126,127]. Patient consent constitutes a fundamental ethical obligation; however, AI systems frequently utilize patient data retrospectively without obtaining explicit consent for particular tasks, thereby raising ethical issues [128]. AI in health care might yield improved therapeutic choices at reduced costs if the technology improves practitioners’ assessment performance. However, it remains unclear if this would incur additional costs. The financial benefit of reducing the expenditure on AI or the unpredictability of its accuracy was modest. However, data on the danger characteristics of the population were more pertinent. Hence, knowledge with sophisticated training is required to use AI in dentistry.

**Figure 8:**
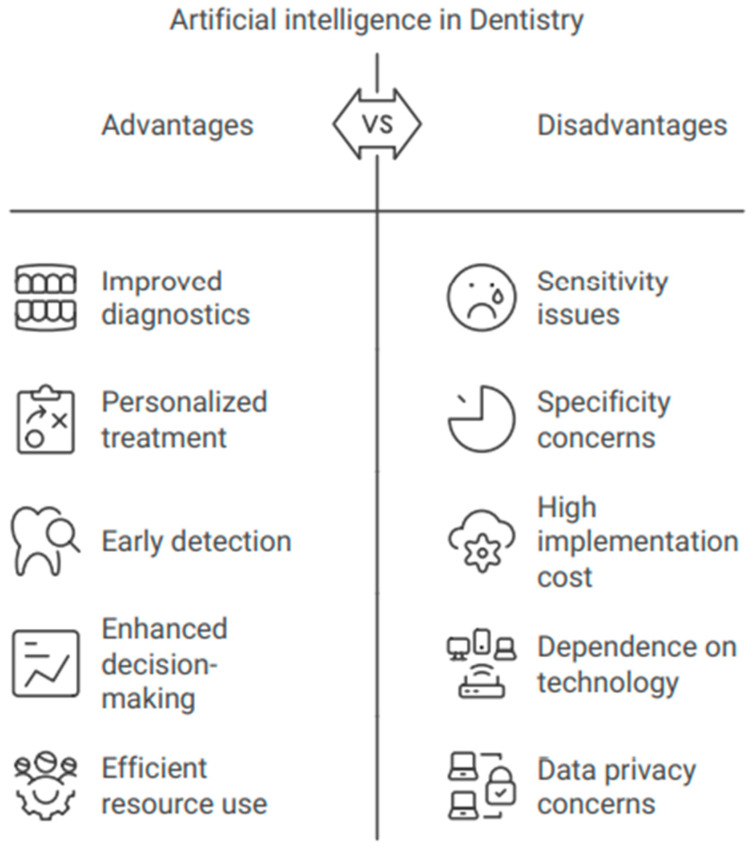
The advantages and disadvantages of artificial intelligence in dentistry.

To maximize economic efficiency, a study into the personalized use of AI for cavity diagnosis appears desirable [20,21,23]. The authors utilized deep-learning techniques centered on convolutional neural networks (CNNs) to identify adolescents who had supernumerary teeth during the initial stages of tooth development. Owing to its accuracy, sensitivity, specificity, and region under the ROC curve, the VGG16-TL prototype performed best, while the additional models also performed well. CNN-based deep learning is a potential method for identifying extra teeth in the initial stages of heterogeneous dentistry. These programs have demonstrated exceptional effectiveness, achieving online accuracy comparable to that of skilled and knowledgeable dental specialists. Dentistry has used these AI-based systems to identify oral plaque, believed to be a precursor to most oral disorders, including tooth decay and periodontal disorders. Patients should not be subjected to the usual approach of finding dental plaque via exploration. An AI strategy based on CNNs can identify oral plaque in a child’s teeth. Compared with a skilled dentist, an AI system showed greater precision in recognizing dental plaque [129]. The field of dentistry has extensively utilized AI technologies. Investigations into the application of AI in dentistry have found that neural networks outperform dental specialists with more reliability and efficiency. In several types of research, AI models have also performed better than experts. AI has advantages and disadvantages that are summarized in Figure 8.

The incorporation of generative AI, especially GPT-like models, in dentistry is an emerging trend that is revolutionizing patient communication and decision-making support [119]. Diagnostics, patient interaction, and instruction, including ChatGPT, utilize these AI models. They can improve patient communication, clinical decision making, healthcare accessibility, and efficacy. However, privacy concerns and the need for training and supervision make these technologies difficult to implement. Generative AI in dentistry requires these components. Generated AI models like ChatGPT can help dentists and patients connect by answering inquiries, scheduling appointments, and providing treatment information. This boosts patient happiness and engagement [130,131]. Personalized health information and reminders from these models can improve treatment adherence [130,131,132]. Surana et al. [133] opined that generative AI helps dentists determine dental restorations and issues on radiographs, improving clinical diagnostics. AI models can evaluate patient data and suggest viable therapy options, especially in complex cases [119,130,132]. To increase critical thinking and problem solving, dental education uses generative AI [134]. This tool provides immediate feedback and simulates clinical situations, preparing trainees for practical applications [131]. It also aids in literature evaluation and discovery, advancing dental knowledge [132]. Nevertheless, we must address data privacy concerns and the overreliance on AI-generated recommendations, given the great potential of generative AI in dentistry. Institutions must set rules and procedures to ensure AI use, emphasizing human control and the critical evaluation of AI outputs. AI apps can assist dentists not only in providing professional advice but also in supplementing and occasionally relieving them of tasks such as incorporating patient information and establishing professional connections [119,130]. AI is proficient at exploiting organized information and extracting inferences from large amounts of data, but it cannot make complex decisions like the human brain can [135,136]. Higher-level comprehension is essential in ambiguous scenarios such as physical examinations, integrating medical histories, appraising esthetic findings, and promoting discourse. Effective patient–dentist engagement necessitates a multimodal assessment of the child’s preferences, concerns, and goals. Despite disagreements about the incorporation of empathy into AI systems, these modes of communication are characterized by impulsivity and illogical behavior. AI can enhance patient care and reduce healthcare system strain by automating routine tasks and allowing clinicians to focus on complex cases. However, ethical principles should guide AI, as it cannot replace human expertise. Despite challenges like data collection, interpretation, and computational power, AI is a valuable tool for dentists due to its unbiased, reproducible, user-friendly, and transparent design. Future AI development should prioritize human interests while improving big-data processing. As dentists is a multidisciplinary field, dentists must make the final decisions. AI is progressing rapidly and has the potential to become a standard tool in dentistry.

## 7. Conclusions

AI is a promising and growing technology in the field of dentistry, and it can reduce dental practitioners’ workloads and improve precision in diagnosis, decision making, treatment planning, and disease prognosis. In reality, AI is just a tool that may be programmed to perform exceptionally well and rapidly. Its successful integration necessitates a safe and controlled integration process, which, in turn, requires dental and continuing education training. AI also plays a critical role in incorporating aspects of these technologies. As various dental disciplines develop AI systems, their future in the healthcare system is promising, offering significant aid to oral health professionals. However, further research is required to more fully adopt AI in the dental field.

## Data Availability

Not applicable.
